# Surgical decisions on implant-related parameters can enhance knowledge transfer for glenoid bone grafting in primary reverse shoulder arthroplasty: a scoping review of heterogeneity sources

**DOI:** 10.1530/EOR-23-0128

**Published:** 2024-10-03

**Authors:** George Mihai Avram, Aleksandra Królikowska, Berte Bøe, Paweł Reichert, Ion-Andrei Popescu, Roland Becker, Robert Prill

**Affiliations:** 1 Department of Orthopedics and Traumatology, Central Military Emergency Hospital Dr. Carol Davila, Bucharest, Romania; 2 Physiotherapy Research Laboratory, University Centre of Physiotherapy and Rehabilitation, Faculty of Physiotherapy, Wroclaw University, Wroclaw, Poland; 3 Division of Orthopedics Surgery, Oslo University Hospital, Norway; 4 Department of Orthopedics, Traumatology and Hand Surgery, Faculty of Medicine, Wroclaw Medical University, Wroclaw, Poland; 5 Orthopedic Surgery and Sports Clinic, ORTOPEDICUM, Bucharest, Romania; 6 Center of Orthopedics and Traumatology, University Hospital Brandenburg/Havel, Brandenburg Medical School Theodor Fontane, Brandenburg a.d.H., Germany; 7 Faculty of Health Sciences Brandenburg, Brandenburg Medical School Theodor Fontane, Brandenburg a.d.H., Germany

**Keywords:** biomechanics, glenoid bone grafting, implant-related parameters, reverse shoulder arthroplasty

## Abstract

**Purpose:**

- The purpose of the present scoping review is to identify sources of heterogeneity in reporting domains that have the potential to improve surgical decision-making in reverse shoulder arthroplasty associated with glenoid bone grafting.

**Methods:**

- A scoping review was conducted according to the JBI and PRISMA ScR guidelines. Articles covering glenoid bone grafting at the time of reverse shoulder replacement, published between 2012 and 2022 in MEDLINE, Scopus, Epistemonikos, Web of Science, and Cochrane Database of Systematic Reviews, were searched. Reporting of core outcome measures, as well as prosthesis-related variables, rehabilitation protocols, radiographic evaluation methods, and bone graft incorporation assessments, were collected.

**Results:**

- For the final analysis, a total of 14 articles were considered. There were 649 operated shoulders with a mean patient age of 72 years, ranging from 48 to 85 years. Analysis of preoperative ranges of motion and patient-reported outcome measures revealed increased heterogeneity in patient selection between studies. Reporting of implant-related parameters was the most inconsistent. Rehabilitation protocols were scarcely reported. Assessment of graft incorporation and radiographic evaluation of choice revealed that there is no consistent method or measurement and the clinical relevance of radiolucent lines at the graft-glenoid bone interface is unclear.

**Conclusion:**

- Addressing implant-related reporting gaps has the highest potential to improve surgical decision-making and provide orthopedic surgeons with a more comprehensive understanding of published results on glenoid bone grafting at the time of primary reverse shoulder arthroplasty.

## Introduction

Recent years have shown us an exponential increase in reverse shoulder arthroplasties (RSA), resulting in an increased overall number of performed replacements (1). The RSA is commonly used to address primary and secondary glenohumeral osteoarthritis, and in many cases, associated glenoid bone defects also need to be addressed (2). There are several bone grafting procedures available, and their effect on the position of the center of rotation (COR) can vary. Depending on the extent of the defect, glenoid bone grafting can be performed to achieve different objectives (3, 4, 5, 6). These objectives include: (i) restoring the native glenoid version and lateral margin with or without extending the COR in cases of the uncontained and severely retroverted glenoid; (ii) grafting a contained glenoid defect with or without lateralizing the COR; or (iii) lateralizing the COR in cases where glenoid bone grafting would not otherwise be necessary to achieve glenoid baseplate stability. It is essential to carefully consider the interface between the native glenoid and bone graft, as well as the interface between the bone graft and the glenoid baseplate, in relation to the COR, regardless of the purpose of the grafting procedure (7). Compression forces should be maximized at each interface throughout the range of motion, while shear forces should be minimized as much as possible (8, 9). The COR position must be considered in order to achieve implant stability and successful graft integration due to its inherent link to moment generation. However, the determinants that impact the position of the COR may not always be apparent (10, 11).

Intraoperatively, the COR’s relationship to the resulting moment arms can be optimized for joint stability by adjusting certain biomechanical variables, depending on the RSA system being used. These variables are located at both the humeral side—stem version, neck-shaft angle, humeral lateralization, humeral cup depth, insert constraint and position—and the glenoid side of the implant—glenoid component version, lateralization, glenosphere size and position, baseplate lateralization, and inferior offset (11). Additionally, bone grafting can be utilized as an independent intraoperative option in order to modify the COR by extending the scapular neck, which laterally shifts the COR without increasing the distance between it and the glenoid bone (12). Thus, glenoid bone grafting not only provides the benefits of a lateralized center of rotation but also does this without increasing shear forces at the bone-glenoid baseplate interface (13).

Despite the available literature on glenoid bone grafting at the time of primary RSA, it has become evident that moderate to substantial heterogeneity is present across multiple studies in terms of ASES and Constant-Murley scores, range of motion, complication, revision, and notching rates (14). The first steps to standardize the reporting of clinical results for RSA have been made by various groups which have developed core outcome sets for uniform reporting standards (15, 16, 17). Nonetheless, increased heterogeneity persists in clinical studies reporting results following RSA and glenoid bone grafting.

The primary aim of this scoping review is to assess the level of detail in reporting implant-related factors in the existing literature related to glenoid bone grafting in Reverse Shoulder Arthroplasty (RSA). The secondary goal is to analyze how data on patient-reported outcome measures, range of motion, graft incorporation, and radiolucencies are reported.

## Materials and methods

### Protocol registration

Prior to the study inception, a research protocol was developed in accordance with the Preferred Reporting Items for Systematic Reviews and Meta-Analyses Extension for Scoping Reviews (PRISMA-ScR) and the JBI Evidence Synthesis Recommendations for Scoping Reviews (18, 19). Once the protocol was finalized, it was published on the Open Science Framework (OSF) before commencing data extraction. To facilitate surgical relevance, three international topic experts from three different European countries were invited to participate and provide their feedback on (i) the relevance of the biomechanical parameters missing from the included studies and (ii) their influence over patient-reported outcome measures, complications, revision, and notching rates, as well as graft incorporation.

### Data sources and searches

An initial search was performed in PubMed to define key concepts focusing on the last 10 years of published literature, between 2012 and 2022. There were three key concepts present in every screened article: i) reverse shoulder arthroplasty; ii) glenoid bone defect; and iii) glenoid bone grafting. After the key concepts were determined, synonyms for each of them were identified by searching within the Similar articles and MeSH term subgroups in order to develop an exhaustive search strategy (see Supplemental Materials, see section on supplementary materials given at the end of this article). Following this step, the search strategy was optimized for each of the databases, including PubMed Central (MEDLINE), Scopus, Epistemonikos, Web of Science, and Cochrane Database of Systematic Reviews, and a complete search of these databases was performed on the 10th of August 2022. Each database was screened for studies published in the last ten years given developments in implant design and surgical technique. Following this step, citations were uploaded into EndNote 20.0.1, and duplicates were removed.

### Study selection

**Figure 1:**
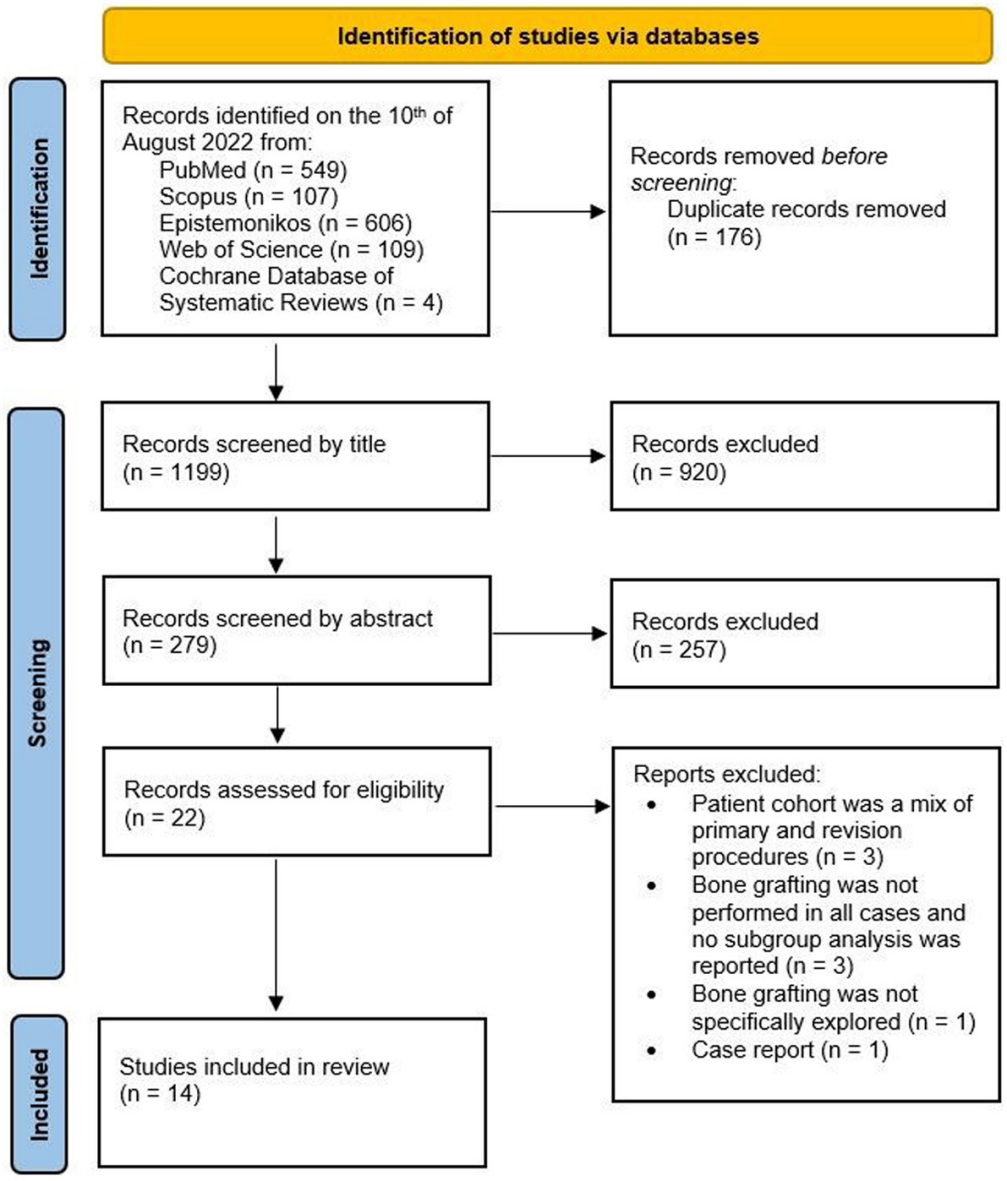
PRISMA flow diagram depicting each step of the conducted study.

Figure 1 provides a detailed explanation of the study selection process. Following the removal of duplicates, the selection process was conducted by two reviewers first by excluding studies based on titles, then on abstracts, and finally following full-text reading. Disagreements were resolved by discussion with a third reviewer.

### Eligibility criteria

During full-text screening, the following inclusion criteria were followed: i) reverse shoulder arthroplasty in a primary setting with associated glenoid bone grafting; ii) complete patient demographics; iii) range of motion report; iv) at least one reported outcome measures; v) English, German, Polish, and Romanian literature to improve generalizability, although this has not been reported to be the case when excluding languages other than English (20). The following exclusion criteria were applied: i) biomechanical simulations; ii) cadaver studies; iii) case reports; iv) studies focused on metal-augmented glenoid baseplates used for treating glenoid bone defects; v) revision cases.

### Quality assessment

No quality, risk of bias, or GRADE assessment of the included studies was performed for this scoping review.

### Data extraction, analysis, and storage

After the full-text screening, 14 articles were included (3, 4, 5, 6, 9, 12, 21, 22, 23, 24, 25, 26, 27, 28). From each included study, data on mandatory reporting domains (pain, physical function/activity, global shoulder function, and adverse events), as well as author, study methodology, and level of evidence, were extracted. Preoperative and postoperative active range of motion, patient-reported outcome measures, implant-related parameters that the authors chose to report in their respective studies, prosthesis design, radiographic assessment of graft integration at follow-up, and rehabilitation protocols were extracted when available to provide a comprehensive picture of the patient’s complete management. For numeric values, arithmetic mean, range, and standard deviation were collected and averaged together to provide single assessments. Each reported complication was counted and reported as a percentage of total complications. String data pertaining to implant selection and reporting, rehabilitation protocols, and radiolucency evaluation were collected in tables and reported as such.

EndNote 20 (Clarivate Analytics, Philadelphia), Microsoft Excel, and Microsoft Word were used for this scoping review.

## Results

Across all included studies, a total of 649 shoulders that underwent RSA and concomitant glenoid bone grafting were identified. The arithmetic mean age of patients was 72.6 years, ranging between 48 and 85 years. The study found that female patients more frequently needed glenoid bone grafting, with 450 females (69%) requiring it, in contrast to 199 males (31%).

Across all studies, the mean follow-up was 39 months, ranging between 23.6 and 78.2 months. Pre- and post-operative ranges of motion and reported outcome measures are reported in Table 1 and Table 2, respectively. The study by Jason Ho *et al*. (25) reported median values of the ASES, SST, and SANE scores and range of motion assessments, and it was not included in either Table 1 or Table 2. Active internal rotation was the most difficult to analyze because some studies report anatomic landmarks (3, 25, 26) that the patient can touch, while others report degrees of movement (22, 24, 28). For this reason, only active internal rotation reported in degrees was collected (Table 1).

**Table 1:** Pre- and post-operative ranges of motion reports.

Motion	Reference	Preoperative values (°)				Postoperative values (°)			
		Mean	s.d.	Min	Max	Mean	s.d.	Min	Max
AAE	(3, 4, 6, 12, 21, 22, 23, 24, 28)	72.2	31.3	10	180	135.5	29.9	20	180
AER	(3, 4, 6, 12, 21, 22, 23, 24, 28)	14.4	18.9	−15	90	26.5	19.3	−15	100
AIR	(22, 24, 28)	2.8	1.6	0*	10*	4.5	1.9	0*	10*
AAb	(3, 5, 6)	25	–^†^	0	60	121.9	–^†^	30	160

AAb, active abduction; AAE, active anterior elevation; AER, active external rotation; AIR, active internal rotation.

°degrees; *reported in a single study (23); ^†^values lacking in all studies (range of preoperative values was provided by Werner *et al*. (6)).

**Table 2:** Pre- and post-operative reported outcome measures.

Score	Reference	Preoperative values				Postoperative values			
		Mean	S.D.	Min	Max	Mean	S.D.	Min	Max
ASES	(3, 21, 22, 28)	30.8	13.2	–	–	74.25	16.1	–	–
Constant	(4, 6, 9, 12, 23, 24)	22.8	12.3	3.7	52	66.4	14.2	32.8	86
SSV	(12, 23, 24, 26)	30.5	15	–	–	81.75	18	20	100
SST	(9, 12, 21, 22, 28)	1.6	0.6	–	–	7.34	3.4	–	–
VAS	(3, 21, 22, 27, 28)	7.5	2.2	6	9	1.3	2.8	0*	2*
SANE	(3)	29.5	–	–	–	87.9	–	–	–

ASES, American Shoulder and Elbow Score; SANE, Single Assessment Number Evaulation; SST, Simple Shoulder Test; SSV, Subjective Shoulder Value; VAS, Visual Analog Scale.

*Values reported by a single study (27).

No study reported complete data on implant type, glenoid baseplate inclination, fixation or peg length, glenoid baseplate, and glenosphere diameters, the humeral stem type and neck-shaft angle, type of humeral tray, or polyethylene liner thickness. Also, there was inconsistent reporting on the type of screws used for the glenoid baseplate and the strategy employed to achieve bone graft compression and baseplate stability. Regardless of this, Supplementary Table 1 provides a detailed presentation of RSA prosthetic designs used in conjunction with glenoid bone grafting and biomechanical parameters as they were reported in each included manuscript.

Only five studies reported their postoperative rehabilitation protocol (3, 4, 5, 6, 24). Nonetheless, rehabilitation protocols were also collected and presented in Table 3, together with a brief description of each study group’s characteristics to have a brief overview of each patient sample heterogeneity.

**Table 3:** Postoperative reported rehabilitation protocols.

Reference	Patient characteristics	Rehabilitation protocol
(24)	Cuff tear arthropathy (*n* = 47) Massive cuff tear (*n* = 20) Failed cuff repair (*n* = 28) Primary osteoarthritis (*n* = 21) Fracture sequelae (*n* = 20) Acute fracture (*n* = 3) Rheumatoid arthritis (*n* = 4) Post-instability osteoarthritis (*n* = 3)	Postoperative rehabilitation after a lateralized BIO-RSA was not altered from that after a standard (medialized) RSA. A sling was worn for 3–4 weeks, and passive ROM was started on postoperative day 1. Pendulum exercises were performed 5 times a day, for 5 min per session. After 4 weeks, formal physical therapy was started, with no heavy lifting until 3 months postoperatively. Return to all types of activities, including gardening, swimming, and golf, was permitted at 3 months postoperatively.
(6)	Neglected anterior glenohumeral dislocation and concomitant rotator cuff deficiency (*n* = 21)	Postoperatively, passive physiotherapy with an abduction splint was prescribed for 6 weeks, followed by active mobilization. Strength exercises were restricted for 3 months postoperatively to protect the bone graft
(5)	3- or 4-part proximal humerus fracture 2-part fracture associated with humeral head splitting 2-part proximal fracture involving the greater tuberosity associated with a history of painful shoulder related to a degenerative long-standing rotator cuff tear All patients sustained a traumatic shoulder dislocation associated with an anterior glenoid rim fracture	Postoperatively, patients were placed in a sling for six weeks. Passive motion with flexion to 90 degrees and external rotation to 30 degrees is started after 3 weeks in the supine position. Active-assisted motion in all planes is initiated starting at week 6.
(3)	Primary glenohumeral osteoarthritis with significant posterior glenoid bone loss and intact rotator cuff (*n* = 29)	`All shoulders were immobilized with a sling and abduction pillow for 4–6 weeks, coming out of the sling 3 times a day to do pendulum, elbow, wrist, and hand exercises. Patients were also allowed to come out of the sling for hygiene. The hand could be utilized for simple tasks; however, no active lifting was allowed. After 4–6 weeks, the sling was discontinued, physical therapy consisting of gradual range of motion and progressive strengthening exercises was initiated, and progression of activities as tolerated was allowed.
(4)	Patient who underwent primary RSA with no glenoid bone loss were included (the research was focused on the effect of lateralizing the center of rotation)	Postoperatively, the arm was placed in a sling for 4 weeks. Passive elevation and external rotation were allowed immediately after the operation. After 4 weeks, the sling was discontinued, and active ROM was initiated. Activities of daily living were progressed, but strengthening was not specifically recommended

A high degree of heterogeneity in reporting standards was noted when analyzing how authors perform the radiographic evaluation of graft incorporation. In this case, heterogeneity is characterized by high variability in evaluation procedures and inconsistencies in what is considered bone graft ‘incorporation’. Data regarding the reporting of glenoid bone graft incorporation, evaluation of radiolucency, and the preferred methodology employed for these assessments are presented in Table 4 for each study included in the analysis.

**Table 4:** Definitions of glenoid bone graft incorporation and radiolucencies.

Reference	Report on bone graft incorporation and/or radiolucencies	Method
(21)	Periprosthetic radiolucency was defined as follows: grade 0 = no radiolucent line, grade 1 = incomplete 1-mm line, grade 2 = complete 1-mm line, grade 3 = incomplete 1.5-mm line, grade 4 = complete 1.5-mm line, and grade 5 = complete 2-mm-wide radiolucent line; Graft incorporation was defined for the purposes of this study as fully incorporated (>75%), partially incorporated (25% to 75%), or not incorporated (<25%) according to the amount of graft remaining on the latest axillary radiographs;	Axillary radiographs
(12)	Aside from the three patients with glenoid loosening, no other patients showed radiographic signs of baseplate loosening such as lucency around the screws or a change in position of the baseplate. Complete incorporation was observed in all patients other than the three with complications. Radiographs and CT images demonstrated union between the cancellous bone graft and the surface of the native glenoid in 94% (51 of 54) of the patients.	Radiographs and postoperative CT scan
(24)	On the glenoid side, radiographs and CT scans were examined for the following: Bone graft healing was defined by the absence of lucent lines observed between the bone graft and the native glenoid. Bone graft viability was assessed based on lysis and/or decreased thickness of the graft. Glenoid component fixation was assessed, and baseplate fixation was graded as stable (no evidence of radiolucency at the baseplate-bone interface or around the peg or any screw), at risk (>1 mm of circumferential radiolucency at the baseplate-bone interface or around the peg or any screw), or loose (either >1 mm of radiolucency around the baseplate-bone interface and around all screws or the existence of a shift in the position of the baseplate).	True anteroposterior radiographs in the plane of the glenohumeral joint and a CT scan
(22)	In 56 cases (98%), the graft was fully incorporated There were 4 major complications (7%) in the study group, and none of them involved glenoid baseplate failure One baseplate demonstrated radiolucent lines concerning for loosening; however, the patient did not show signs of clinical failure and therefore did not undergo revision surgery.	Postoperative radiographs included (anteroposterior, Grashey, scapular Y, and axillary lateral)
(23)	When osteolysis of the inferior part of the graft was observed, the images were independently reviewed by a second investigator to differentiate between classical graft osteolysis (oblique erosion) and notching (double contour erosion) The radiographs were also assessed for radiolucent lines around the post and screws and for any other obvious signs of glenosphere loosening or component disassembly. Radiolucencies >2 mm in width were considered radiological signs of loosening	AP radiographs of the glenohumeral joint on three views (shoulder in internal rotation, neutral, and external rotation)
(25)	Graft resorption was determined as a change in the cortical borders of a graft in relation to the baseplate compared to the early postoperative radiograph	True AP (Grashey) and axillary views
(26)	Periprosthetic radiolucency was defined as follows: grade 0, no radiolucent line; grade 1, incomplete 1-mm line; grade 2, complete 1-mm line; grade 3, incomplete 1.5-mm line; grade 4, complete 1.5-mm line; grade 5, 2-mm-wide lucent line and complete A glenoid component was considered to be ‘at risk’ for clinical loosening if there was migration or tilt of the component or glenoid lucency of grade 4 or higher Glenoid graft resorption was quantified from 0% to 100%.	Anteroposterior view in internal and external rotation and an axillary view
(6)	Radiographic examination focused on: bone graft resorption heterotopic ossification glenoid component loosening	Standardized anteroposterior view in neutral rotation with the arm hanging by the side and an axillary view with the patient in the supine position with the arm abducted
(9)	Radiographic examination focused on: progressive radiographic lucent lines progression of notching loosening of the baseplate loosening proximal humeral bone loss bone graft incorporation the initial postoperative radiograph was compared with the radiograph taken at the most recent visit, and graft incorporation and radiolucent lines were noted	AP radiographs
(5)	Radiographic examination focused on: baseplate displacement or loosening bone graft union resorption or collapse	Anteroposterior view in neutral rotation and Bernageau view
(3)	Radiographic evaluation was focused on: autograft incorporation autograft resorption baseplate loosening posterior instability scapular notching	Two grashey (with humerus in internal and external rotation), scapular-Y, and axillary views.
(4)	Postoperative radiographs were assessed for bone graft incorporation defined by: the absence of lucent lines observed between the humeral bone graft and the native glenoid inferior notching at the native glenoid radiolucent lines (around the peg, screws, and humeral stem) shift in the position of the components. The severity of the inferior notching was graded according to the Sirveaux classification. Analysis of the superior aspect of the bone graft is difficult because the coracoid process often overlaps the superior aspect of the joint line. We therefore focused on the inferior aspect of the graft, below the level of the central peg. For the BIO-RSA cohort, inferior graft incorporation was also graded according to the system of Boileau et al.(44)	Standardized radiographs in anteroposterior in neutral, external and internal rotation, and axillary lateral views
(28)	Postoperative radiographs were evaluated for: incorporation and resorption of the bone graft migration or subsidence of the baseplate scapular notching as graded using the Nerot–Sirveaux system.	Anteroposterior, Grashey anteroposterior, scapular-Y lateral, and axillary lateral views

**Figure 2:**
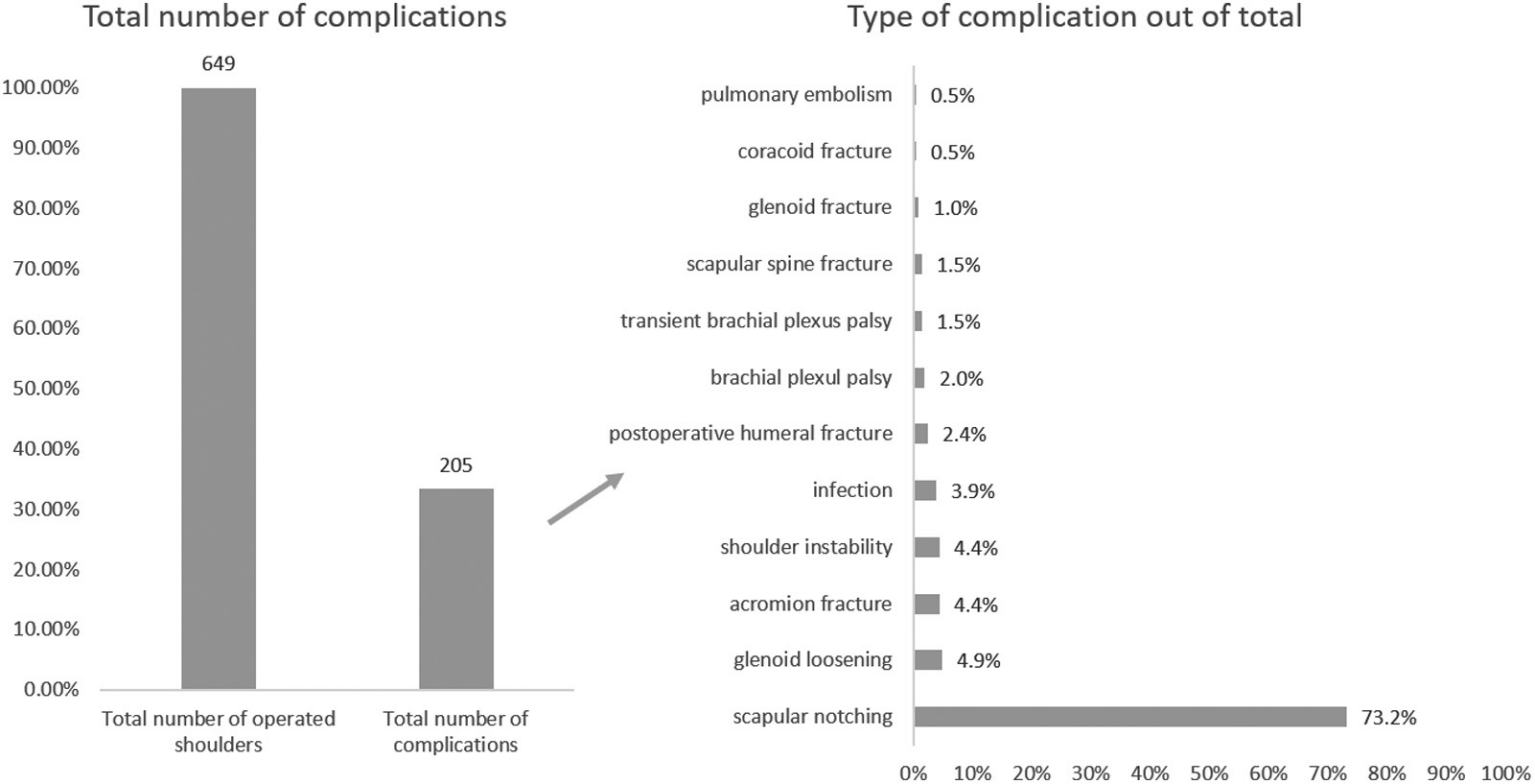
Total number of reported complications. Out of a total of 649 operated shoulders, there were 205 reported complications representing a 32% overall complication rate with scapular notching contributing to 73% of all complications.

**Figure 3:**
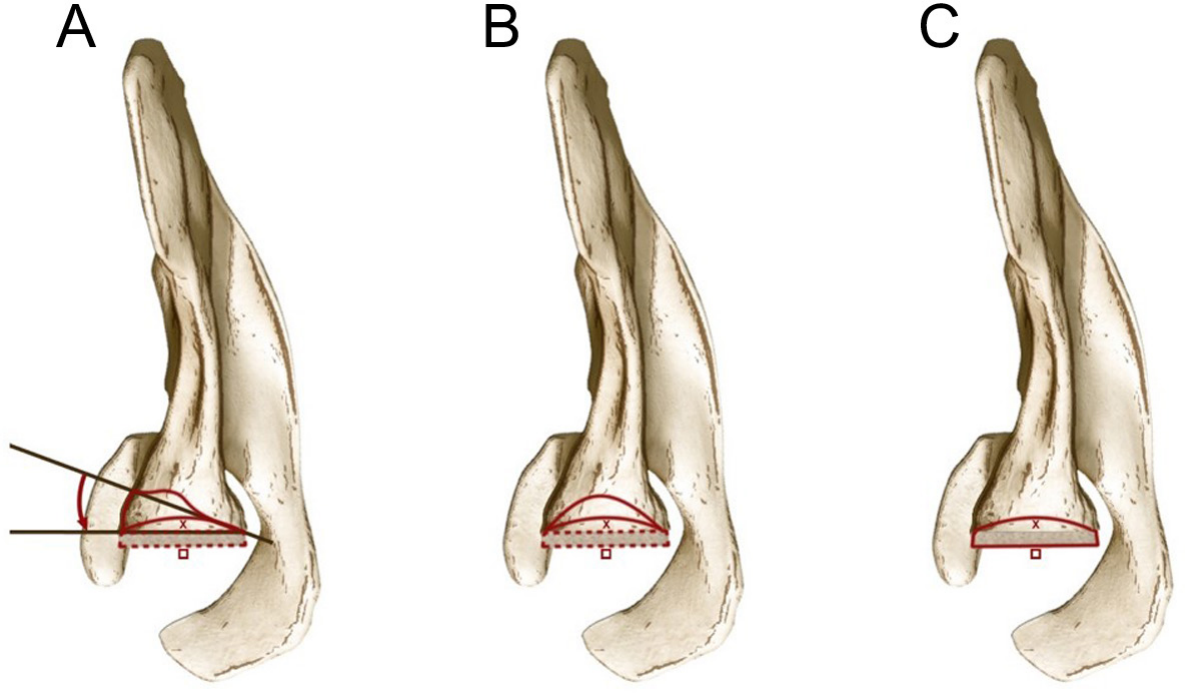
Drawing of the three situations, as viewed in the axial plane, in which glenoid bone grafting was performed in the included studies. (A) Glenoid version correction (curved red arrow) and lateralization (red square) of the COR from its native position (red x mark) in situations of uncontained glenoid bone defects; (B) Glenoid bone grafting (continuous red line) and lateralization of the COR (red square) from its native position (red x mark) in cases of contained glenoid defects; (C) Glenoid COR lateralization (red square) from its native (red x mark) in patients with minimal glenoid bone erosion.

**Figure 4:**
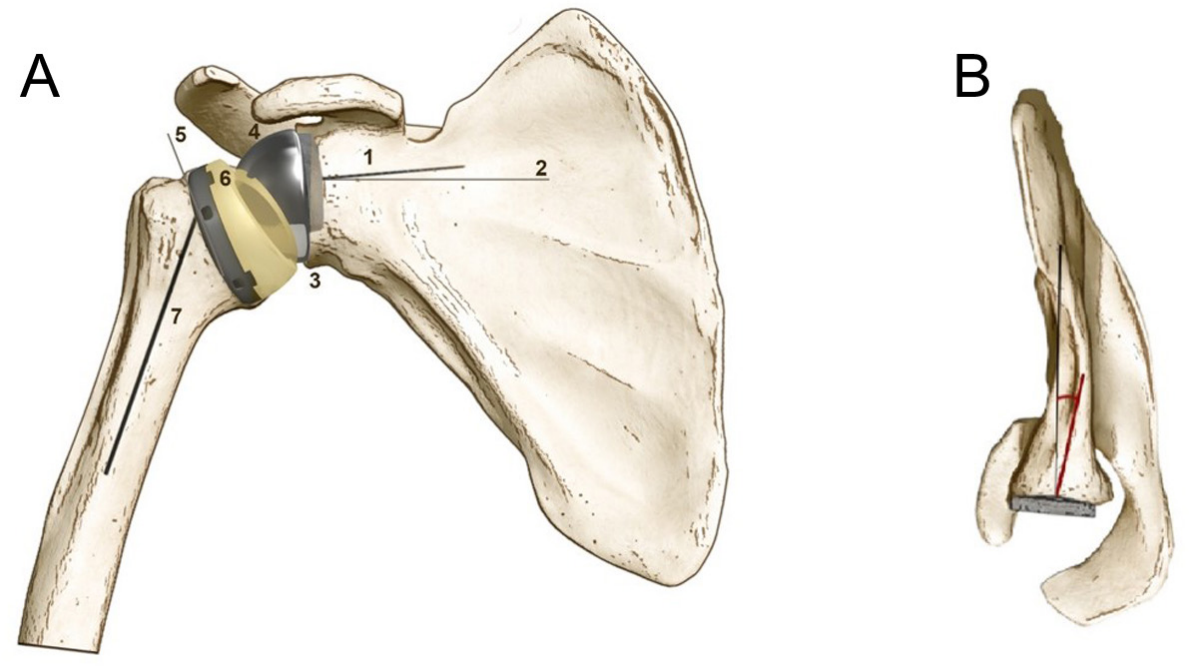
Basic implant-related parameters pertaining to reversed shoulder arthroplasty in the coronal and axial plane. (A)1. Glenoid baseplate peg length; 2. Glenoid baseplate tilt in relation to the supraspinatus fossa as described by (41); 3. Glenoid baseplate diameter; 4. Glenosphere diameter; 5. Humeral neck cut angle; 6. Polyethylene tray type (inlay/onlay) and thickness; 7. Humeral stem type in relation to the anatomical axis of the humerus as described by (11). (B) Glenoid baseplate version in relation to Friedman’s line.

**Figure 5:**
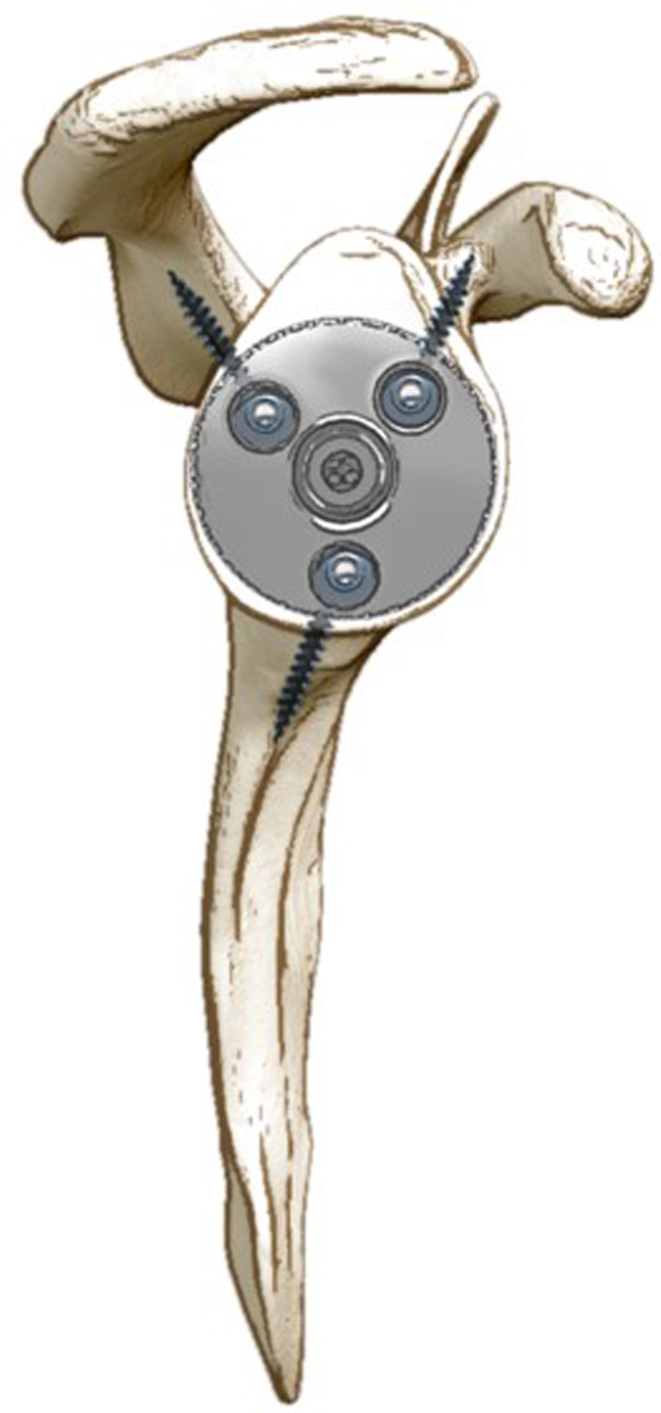
Scapular Y view of the glenoid baseplate. In order to maximize screw purchase and baseplate compression, the three-column concept, as was presented by (43) can be applied using a variable-angle glenoid baseplate.

Reported adverse events were recorded and summarized in Fig. 2. There were 14 reported revisions with 5 (36%) due to glenoid loosening, 4 (29%) due to persistent shoulder instability, 3 (21%) for infection, and 2 (14%) for periprosthetic humeral fracture, respectively (12, 23, 25). Overall, there were three main reasons reported for bone grafting the glenoid during primary RSA: i) to lateralize the center of rotation; ii) to graft a glenoid defect or to restore version and achieve glenoid baseplate stability; iii) to both lateralize the center of rotation and graft a glenoid defect and restore glenoid version (Fig. 3). In order to simplify the reporting of implant-related parameters, based on what has been presented in the included articles, the present paper summarizes key implant-related parameters that should be reported in future studies in order to enhance transparency and provide readers with a better understanding of postoperative results (Table 5, Figs 4 and 5).

**Table 5:** General recommendations of implant-related variables to be reported in future studies.

Biomechanical parameter to be reported	Reasoning behind its relevance
1. Glenoid component baseplate diameter and tilt	There are multiple reverse shoulder systems in which the glenoid baseplate size determines the available glenosphere options. Furthermore, the tilt of the glenoid baseplate is relevant because it influences the center of rotation location, peg length, as well as the bone purchase of the baseplate screws (Fig. 5).
2. Glenosphere diameter and type	Several reverse shoulder systems available provide standard, inferior-offset, and lateralized glenospheres.
3. Glenoid baseplate fixation mechanism	Especially when radiolucency and revision rate are of interest, it is preferable that authors report central peg diameter and length together with the type of associated baseplate screws (locking, non-locking, angular-locking, compression-angular-locking, etc.).
4. Purpose of glenoid bone grafting	As underlined in Fig. 3, glenoid bone grafting can be utilized in multiple situations and for different reasons. Therefore, in order to better understand the outcomes of surgery, readers should be informed on the purpose of glenoid bone grafting.
5. Type of humeral component	Long, straight stems have a tendency to decrease the deltoid lever arm more so than short stems or stemless stems. In turn, a decreased deltoid lever arm influences the stability of the implant, which would determine the surgeon to utilize a more constrained or thicker humeral bearing.
6. Humeral bearing surface thickness and offset	Reverse shoulder systems can provide the option to adjust the offset of the humeral bearing surface. This, in turn will provide the surgeon with the option to optimize soft tissue tensioning and the resulting range of motion.

## Discussion

The primary finding of this scoping review was that every study reported mandatory core outcome domains. However, clear inconsistencies are apparent in terms of implant-related parameter reporting, ranging from studies that did not report them (21) to those that provided a clear description of the entire decision process (23, 24). The most significant variation among studies was observed in the utilization of different RSA designs, as illustrated in Supplementary Table 1. While this variability was expected by the author group, it presents a challenge when attempting to draw conclusions that could enhance surgical knowledge. This is because each prosthetic design manages the center of rotation by manipulating prosthetic parameters to different extents, which in turn can have varying weight on joint stability and postoperative passive and active range of motion (10, 11). From this perspective, it is logical to assume that patient-reported outcome measures and adverse events may be influenced by prosthetic design and surgical decision-making. This perspective is underlined, especially when discussing adverse events, as scapular notching was identified to comprise up to 73% of all complications (Fig. 2). The procedure of bone grafting on the glenoid side to laterally adjust the center of rotation has been reported to reduce the incidence of notching, the most frequently reported adverse event following RSA (12). The potential of reducing the rate of scapular notching is especially emphasized with the use of an angled bone graft that can lateralize and provide the glenoid baseplate with inferior tilt while at the same time correcting posterior glenoid wear (12). The relevance of this bone grafting technique comes from its ability to prevent medialization when the glenoid baseplate is tilted inferiorly. When combined with a medialized prosthetic design or a neck-shaft angle (NSA) of 155°, the effect of laterally extending the scapular neck by bone grafting could be nullified, thereby limiting the evaluation of the potential beneficial impact of glenoid bony lateralization. Simultaneously, it is noteworthy that on the humeral side, lateralization can be achieved to a greater extent than on the glenoid side (11). Long and short humeral stems exhibit varying degrees of lateral displacement of the humerus, while in stemless implants, lateralization is controlled at the level of the humeral cut (10). However, even with various choices for lateralizing the center of rotation, notching remained the most frequently reported complication, prompting the question of what measures surgeons can effectively take to reduce notching. In terms of adaptability, inlay designs offer less flexibility in the sagittal plane. In contrast, onlay designs can be adjusted to modify the lever arms of the anterior or posterior rotator cuff based on intraoperative findings (10). NSA is commonly recognized as a major contributor to adverse events like notching at the inferior scapular neck (29). Decreasing its value from 155° to 145° or even 135° has the potential to reduce notching rates while at the same time leaving behind the question of potential instability, which is difficult to determine due to multiple identified confounders (30). Therefore, there is no ideal implant and surgeons should weigh the contribution of implant biomechanics as well as soft-tissue management in order to optimize stability and range of motion.

The choice of one outcome score over the other or assessing postoperative ranges of motion can be seen in Tables 1 and 2. Although reaching minimal clinically important difference (MCID) by SANE score is correlated with achieving MCID by Constant score, it is unknown how patient characteristics in the case of Constant score influence this correlation as its values tend to decrease with age and female gender (31, 32). This is especially relevant in the case of reverse shoulder replacement associated with glenoid bone grafting as, in general, this population is represented by older female patients, as pointed out by present findings. The American Shoulder and Elbow Society (ASES) score was the second most commonly employed scoring system, and although it has been criticized for being overly inclusive, its use for assessing patient outcomes following reverse shoulder replacement has been validated (32, 33). Using the Simple Shoulder Test (SST) needs careful consideration as MCID is different depending on the arthroplasty type, and in the case of RSA, a value of 3.7 has been reported (34). The Subjective Shoulder Value (SSV) score, although considered easy to interpret, could be considered one of the most subjective measures of the ones identified. The correlation analyses conducted using the Constant score have indicated that the SSV score might have potential application in patients experiencing osteoarthritis or instability (35). Interestingly, in these cases, the SSV score displayed a weak correlation with the Constant score, while a stronger correlation with patient arm function was observed in relation to the Constant score rather than the SSV score (36). Choosing structurally valid and reliable patient-reported outcome measures ensures relevant and trustworthy follow-up.

Glenoid defect assessment during preoperative evaluation for primary reverse shoulder replacement has proven difficult to condense into a single comprehensive classification. This aspect is proven by current classification systems that are helpful in defining glenoid defects in single anatomical planes (37, 38, 39, 40). The present scoping review has identified increased heterogeneity in the assessment of glenoid bone graft and baseplate integration at postoperative evaluations, as well as in the evaluation of radiolucencies (Table 4). There seems to be a lack of consensus regarding the preferred method (roentgenographic or computed tomography scan) or the optimal approach for assessing these variables. Additionally, there is no clear agreement on how to evaluate radiolucencies or their clinical significance in relation to surgical decision-making. Nonetheless, significant progress is being made toward determining the measurement accuracy between radiographic and computed tomography scans in determining glenoid inclination for preoperative planning (41). Such reports must be included in future recommendations to provide a comprehensive guide for preoperative planning that can potentially guide intraoperative decision-making. Furthermore, due to the lack of any recommendations or consistent approaches, surgeons should rely on patient symptoms and relevant clinical signs when dealing with radiolucencies and their potential impact on implant stability.

It is crucial to acknowledge that the presence of these various sources of heterogeneity prevents the possibility of conducting a systematic review and meta-analysis. Therefore, any conclusions drawn from the included studies should be approached with caution. It is worth noting that all studies were conducted by leading shoulder surgeons, and clinicians should be mindful that the reported outcomes of glenoid bone grafting in combination with reverse shoulder replacement may not be replicable in their own practice. In addition, nearly every study included in the analysis reported the use of a different reverse shoulder system (Supplementary Table 1), with limited guidance on how to effectively utilize the system's design principles to achieve stable graft and glenoid baseplate fixation, as well as optimal passive and active shoulder range of motion. The heightened heterogeneity in implant design has been recognized by previous studies as a significant limitation that hinders the establishment of standardized reporting criteria (42). Consequently, the recommendations provided in Table 5 facilitate future research focusing on biomechanical parameters and their link to postoperative results following RSA in greater detail. The present scoping review has highlighted that from preoperative evaluation to postoperative assessment measures, studies focusing on glenoid bone grafting at the time of primary reverse shoulder replacement are dominated by increased heterogeneity. The primary factor contributing to the observed heterogeneity is the variation in the purpose of glenoid bone grafting and the specific way in which each prosthetic parameter is manipulated during surgery to obtain graft compression and improved stability. For this reason, the recommendations provided in Table 5 might help authors and readers alike enhance the transparency of reporting and improve decision-making in the future. Because previous studies have underlined many differences in reverse shoulder replacement design philosophy, the present scoping review advocates, through the provided recommendations, for more transparent reporting of intraoperative handling of biomechanical parameters.

### Limitations

Clinicaltrials.gov interrogation and a second, more recent search were not performed due to time constraints.

## Conclusion

Glenoid component baseplate diameter and tilt, glenosphere diameter and type, glenoid baseplate fixation mechanism, purpose of glenoid bone grafting, type of humeral component, and humeral bearing surface thickness and offset are recommended as relevant implant-related factors that should be reported in future studies focusing on RSA and glenoid bone loss in order to enhance transparency and influence future intra-operative decision-making. Inferior glenoid notching remains the major postoperative complication following glenoid bone grafting during primary RSA despite reported technical improvements. Uncertainties regarding the clinical relevance of glenoid radiolucencies, preferred method of investigation, and progression over time are still debated; therefore, clinical examination and patient complaints remain the most important factors influencing surgical decision-making in these cases.

## Supplementary Materials

Supplementary Table 1: Reported implant related parameters and prosthetic designs

Supplemental Material

## ICMJE Conflict of Interest Statement

The authors declare that there is no conflict of interest that could be perceived as prejudicing the impartiality of the study reported.

## Funding Statement

This work did not receive any specific grant from any funding agency in the public, commercial, or not-for-profit sector.
