# Supplementary material for: Surgical decisions on implant-related parameters can enhance knowledge transfer for glenoid bone grafting in primary reverse shoulder arthroplasty: a scoping review of heterogeneity sources
Source: EFORT Open Rev. 2024 Oct 3;9(10):990–1001. doi: 10.1530/EOR-23-0128 (PMC11457808; doi:10.1530/EOR-23-0128)
Supplement: Supplementary Table 1: Reported implant related parameters and prosthetic designs [file EOR-23-0128supplementary_table_1.pdf]

Supplementary Table 1: Reported implant related parameters and prosthetic designs

Reference	Variable	Prosthesis design used
(1)	-	1. Aequalis Reversed shoulder arthroplasty (Tornier)<br>2. Delta Xtend (DePuy Orthopaedics)
(2)	1. Long glenoid baseplate peg (25, 30, 35 mm)<br>2. Superior and inferior non-locking screws for compressing the graft<br>3. Anterior and posterior locking convergent locking screws	1. Aequalis reversed prosthesis (Wright-Tornier)
(3)	1. Long glenoid baseplate peg (25 mm)<br>2. Superior and inferior divergent screws for compression – exchanged later for locking screws	1. Aequalis (Tornier, Saint-Ismier Cedex, France)
(4)	1. Medialized center of rotation<br>2. 155° neck-shaft angle<br>3. Long central peg (25 mm)<br>4. Glenoid baseplate diameter of 29 mm<br>5. Bone graft thickness of 10 mm for 36 mm glenosphere diameter<br>6. Bone graft of 7 mm for 42 mm glenosphere diameter<br>7. 10° of inferior tilt applied to the glenoid baseplate<br>8. Superior and inferior non-locking screws for compression<br>9. Anterior and posterior convergent locking screws<br>10. Once the glensphere was implanted stability, tension, and range of motion were assessed using a trial humeral prosthesis using a standard +6 polyethylene cup<br>11. Humeral component was implanted with 20°of retroversion	1. Aequalis Reverse Shoulder Prosthesis (Tornier-Wright, Bloomington, MN, USA)
(5)	1. Glenosphere size was determined on the basis of the soft-tissue tension<br>2. The 36-mm and 40-mm glenosphere sizes (in 40 and 5 patients, respectively) offered a hooded design and could increase contact surface area of the glenosphere with the native scapula, and in many cases this design feature was utilized during surgery. However, 32-mm glenospheres (in 12 patients) were used at the discretion of the senior author in the instances in which it best fit the patient’s anatomy.<br>3.	1. Reverse Shoulder Prosthesis; DJO Surgical
(6)	1. All patients received a 135° neck-shaft angle onlay humeral stem, glenoid component lateralization with a humeral head bone graft, and an eccentric glenosphere<br>2. The goal of the glenoid lateralization was to create 5 mm of lateralization between the glenoid component and the native glenoid bone at the level of the post of the baseplate<br>3. Optimization of the implant components was	1. Aequalis Ascend Flex (Tornier, Montbonnot, France)

	planned according to their size (diameter of the base plate and of the sphere), and the characteristics of the bone graft (25 or 29 mm diameter, 0° or 12° angle, and thickness). The objectives were to optimize the size and inclination of the glenosphere to ensure neutral tilt and version while increasing glenoid bone stock and lateralizing the COR. To prevent the polyethylene humeral cup abutting against the scapular neck, the base plate was placed flush against the inferior and posterior border of the glenoid after reaming.<br>4. The humeral head was cut at its anatomical neck following natural retroversion<br>5. Trial trays were used in position 6 (the most lateral tray position) to prevent excessive humeral lateralization, with 1.5-mm or 3.5-mm offsets.<br>6. On the glenoid side, a guidewire was placed to follow the preoperative plan with either a standard or a patient-specific glenoid guide and to place the glenoid base plate flush with the inferior part of the glenoid.<br>7. A long peg (25 mm) glenosphere baseplate was impacted with the bone graft. The glenosphere was fixed with three or four screws based on the available glenoid bone stock and the surgeon's preference.<br>8. A 36-, 39-, or 42-mm eccentric glenosphere was used to increase the inferior offset by 2mm.<br>9. The diameter of the base plate (28 baseplates of 25 mm and 51 baseplates of 29 mm) and the diameter of the glenosphere (36, 39, or 42 mm) were planned preoperatively for each patient and were not randomized.<br>10. The humeral component with a 135° neck-shaft angle (a 127.5° stem, a 1.5- or 3.5-mm offset tray, and a 6-mm thick, 7.5° angled polyethylene cup in the lateral position [position 6]) was placed in the standard fashion.	
(7)	1. Baseplates with a long-post/ screw option were used for all cases. Baseplates with a screw option all had a minimum 35-mm central screw placed<br>2. All peripheral screws in the baseplate were locking.<br>3. Glenosphere size and offset were determined based on surgeon preference and implant stability	1. Trabecular Metal Reverse Total Shoulder Arthroplasty (Zimmer Biomet, Warsaw, IN, USA)<br>2. Aequalis Reversed II Post and Threaded (Wright Medical, Bloomington, MN, USA)<br>3. Delta Xtend Reverse Shoulder System (DePuy Synthes–Johnson & Johnson, Warsaw, IN, USA)<br>4. Titan Reverse Shoulder System (Integra LifeSciences, Plainsboro, NJ, USA)

		5. AltiVate Reverse (DJO Surgical, Austin, TX, USA)
(8)	1. To obtain secure fixation of the implant and bone graft, at least 2 of the glenoid baseplate screws were placed to capture the medial cortex of the scapular neck<br>2. A lateral offset glenosphere was implanted in 6 (15%) shoulders, including the Comprehensive (+3mm offset) and the Encore (+4 mm offset) design. The remaining 35 (85%) implants had a medial center of rotation (Comprehensive and Delta Xtend; no offset)	1. Comprehensive Reverse Shoulder Prosthesis (Biomet, Warsaw, IN, USA)<br>2. Delta Xtend (DePuy Orthopedics, Warsaw, IN, USA)<br>3. Encore Reverse Shoulder (DJO Surgical, Austin, TX, USA)
(9)	1. Lengthened 25-mm pegged or custom-made glenoid baseplate with anchorage length of the central peg in the native glenoid of at least 10mm. Hence, the positioning for the central peg was in some cases oriented slightly divergent from the standard glenoid center line. It did not necessarily pass through the center of the glenoid surface.<br>2. On the basis of preoperative imaging and intraoperative findings, the surgeon selected the peg length and diameter of the glenoid baseplate<br>3. It was determined that the backside of the glenoid baseplate should cover at least half of the width of the native glenoid surface. Therefore, a baseplate with a smaller diameter (25 mm) was inserted if the glenoid bone defect extended to more than two-thirds of the glenoid width<br>4. Drilling was oriented at most in 10° to 15° of anteversion to increase anchorage length in the native scapula	1. Delta reverse shoulder prostheses (DePuy Orthopedics, Warsaw, IN, USA<br>2. Aequalis reversed shoulder prostheses (Tornier, Saint-Ismier, France)
(10)	1. Compression fixation of the graft by the baseplate was attained. A standard (25-mm peg) implant was used	1. Delta III (DePuy International Ltd., Leeds, UK)<br>2. Delta Xtend (DePuy, Warsaw, IN, USA)
(11)	1. A 25 mm length post was used in all cases to ensure the length of the central post anchored in the native glenoid was at least 10 mm.<br>2. In all cases a 25 mm diameter baseplate was implanted<br>3. Fixation was performed using 4 screws. Compression screws were used in the anterior and posterior holes. The anterior compression screw was used to fix the anterior part of bone graft to the scapular neck. Locking screws were used in the superior and inferior holes.<br>4. Once the baseplate was fixed, the glenosphere was then implanted in a standard fashion.	1. Aequalis™ Reversed II<br>2. Ascend flex reverse shoulder arthroplasty (Tornier, Edina, MN)
(12)	1. A long post baseplate was used<br>2. Compression was achieved using two peripheral compression screws, and two peripheral locking screws<br>3. Fifteen size 42mm and 14 size 36mm glenospheres were implanted<br>4. 6mm polyethylene insert was placed in 26	1. Aequalis (Tornier, Edina, MN, USA)

	shoulders. Two shoulders required a 9mm insert, and 1 required a 12mm insert<br>5. All implanted prostheses were based on Grammont principles and included 28 press-fit Aequalis adjustable reversed prostheses and 1 cemented Aequalis reversed prosthesis (Tornier, Edina, MN, USA<br>6. All humeral head cuts were performed at 155° of inclination and 30° of retroversion.<br>7. Guide wire had 0–10° of inferior tilt, and approximately 14 mm superior to the inferior border of the glenoid<br>8. All humeral components were implanted in 30° of retroversion	
(13)	1. A 25 mm long post glenoid baseplate was used<br>2. A 29-mm Aequalis Reversed circular baseplate was implanted at the inferior edge of the glenoid surface<br>3. A centered 36-mm glenosphere with a center of rotation at the glenoid surface was placed over the baseplate<br>4. All humeral stems had a neck-shaft angle of 155° and were cemented after insertion of a cement restrictor plug	1. Aequalis Reversed shoulder prosthesis system (Wright Medical, Montbonnot, France)
(14)	1. The typical thickness of the cut head was between 15 mm and 20 mm and the proximal humerus was cut between 0° and 10° of retroversion in all cases<br>2. The glenoid baseplate guiding pin was positioned to achieve neutral or inferior tilt on a standing true anteroposterior radiograph and between 0° and 10° of retroversion on an axillary radiograph<br>3. The central baseplate post was 25 mm long in all cases, achieving at least 5 mm in native glenoid.<br>4. Once the baseplate was secured, an additional 2.7-mm cortical screw was often placed superior to the baseplate from the graft into the glenoid	1. Trabecular Metal Reverse Total Shoulder Arthroplasty (Zimmer, Warsaw, IN, USA)<br>2. Aequalis Reversed Shoulder Arthroplasty (Tornier, Bloomington, MN, USA)

References

1. Imai S. A Sequential Approach to the Management of Posterior Glenoid Defects in RSA: Angulated BIO Versus Multiple Bioresorbable Pinning-Assisted Structural Bone-Grafting. JB JS Open Access. 2021; 6(4):e21.00049

2. Boileau P, Morin-Salvo N, Gauci MO, Seeto BL, Chalmers PN, Holzer N, Walch G. Angled BIO-RSA (bony-increased offset-reverse shoulder arthroplasty): a solution for the management of glenoid bone loss and erosion. J Shoulder Elbow Surg. 2017; 26(12):2133-2142

3. Bateman E, Donald SM. Reconstruction of massive uncontained glenoid defects using a combined autograft-allograft construct with reverse shoulder arthroplasty: Preliminary results. J Shoulder Elbow Surg. 2012; 21(7):925-34

4. Boileau P, Morin-Salvo N, Bessière C, Chelli M, Gauci MO, Lemmex DB. Bony increased-offset-reverse shoulder arthroplasty: 5 to 10 years' follow-up. J Shoulder Elbow Surg. 2020; 29(10):2111-2122

5. Lorenzetti A, Streit JJ, Cabezas AF, Christmas KN, LaMartina J, Simon P, Frankle MA. Bone Graft Augmentation for Severe Glenoid Bone Loss in Primary Reverse Total Shoulder Arthroplasty: Outcomes and Evaluation of Host Bone Contact by 2D-3D Image Registration. JB JS Open Access. 2017;2(3):e0015

6. Collotte P, Gauci MO, Vieira TD, Walch G. Bony increased-offset reverse total shoulder arthroplasty (BIO-RSA) associated with an eccentric glenosphere and an onlay 135° humeral component: clinical and radiological outcomes at a minimum 2-year follow-up. JSES Int. 2022; 6(3):434-441

7. Ho JC, Thakar O, Chan WW, Nicholson T, Williams GR, Namdari S. Early radiographic failure of reverse total shoulder arthroplasty with structural bone graft for glenoid bone loss. J Shoulder Elbow Surg. 2020; 29(3):550-560

8. Ernstbrunner L, Werthel JD, Wagner E, Hatta T, Sperling JW, Cofield RH. Glenoid bone grafting in primary reverse total shoulder arthroplasty. J Shoulder Elbow Surg. 2017; 26(8):1441-1447

9. Werner BS, Böhm D, Abdelkawi A, Gohlke F. Glenoid bone grafting in reverse shoulder arthroplasty for long-standing anterior shoulder dislocation. J Shoulder Elbow Surg. 2014; 23(11):1655-61

10. Gupta A, Thussbas C, Koch M, Seebauer L. Management of glenoid bone defects with reverse shoulder arthroplasty-surgical technique and clinical outcomes. J Shoulder Elbow Surg. 2018; 27(5):853-862

11. Garofalo R, Brody F, Castagna A, Ceccarelli E, Krishnan SG. Reverse shoulder arthroplasty with glenoid bone grafting for anterior glenoid rim fracture associated with glenohumeral dislocation and proximal humerus fracture. Orthop Traumatol Surg Res. 2016; 102(8):989-994

12. Harmsen S, Casagrande D, Norris T. "Shaped" humeral head autograft reverse shoulder arthroplasty: Treatment for primary glenohumeral osteoarthritis with significant posterior glenoid bone loss (B2, B3, and C type). Orthopade. 2017; 46(12):1045-1054

13. Collin P, Liu X, Denard PJ, Gain S, Nowak A, Ladermann A. Standard versus bony increased-offset reverse shoulder arthroplasty: a retrospective comparative cohort study. J Shoulder Elbow Surg. 2018; 27(1):59-64

14. Tashjian RZ, Granger E, Chalmers PN. Structural glenoid grafting during primary reverse total shoulder arthroplasty using humeral head autograft. J Shoulder Elbow Surg . 2018; 27(1):e1-e8
