# Supplementary material for: Surgical decisions on implant-related parameters can enhance knowledge transfer for glenoid bone grafting in primary reverse shoulder arthroplasty: a scoping review of heterogeneity sources
Source: EFORT Open Rev. 2024 Oct 3;9(10):990–1001. doi: 10.1530/EOR-23-0128 (PMC11457808; doi:10.1530/EOR-23-0128)
Supplement: Supplemental Material [file EOR-23-0128supplementary_material.pdf]

Search strategy
The search strategy will be constructed based on three concepts:
1) Reverse shoulder arthroplasty
2) Glenoid bone defect
3) Glenoid bone defect grafting

The authors have conducted a preliminary data search and are providing the following keywords that will be used to construct the final search:

- **Concept 1:** reverse total shoulder arthroplasty, RTSA, **reverse** shoulder arthroplasty, rTSA, reverse TSA, reverse shoulder prosthesis/prostheses, reverse conversion, reverse shoulder replacement, reverse arthroplasty
- **Concept 2:** glenoid types, Walch _ glenoid*, glenoid bone loss, glenoid bone defect, posteriorly worn glenoid*, anteriorly worn glenoid*, superiorly worn glenoid*, bony defect, posterior bone loss, anterior bone loss, superior bone loss, glenoid deformity, uncontained glenoid (bone) defects, glenoid inclination, glenoid retroversion, glenoid (bone) deficiency, glenoid erosion, glenoid reconstruction, glenoid dysplasia, posterior glenoid deficiency, glenoid morphology, biconcave glen*, Favard type _ glen*
- Concept 3: autograft, allograft, structural allograft, iliac crest autograft, humeral head autograft, structural glenoid grafting, glenoid bone grafting, primary glenoid bone grafting, primary bone grafting, femoral head allograft, femoral allograft, glenoid reconstruction, structural bone graft, glenoid augmentation, bony increased-offset-reverse shoulder arthroplasty (BIO-RSA), structural glenoid allograft, J-shaped bone graft, bony glenoid augmentation

The following databases will be searched:
1) MEDLINE via Pubmed
2) Scopus
3) Epistemonikos
4) Web of Science
5) Cochrane Database for Systematic Reviews
6) Clinicaltrials.gov

The authors are reporting the following preliminary search strategy for each searched database:

- MEDLINE

("Arthroplasty, Replacement, Shoulder"[Mesh] AND reverse) OR ("Shoulder Prosthesis"[Mesh] AND reverse) OR (reverse total shoulder arthroplasty) OR (RTSA) OR (reverse shoulder arthroplasty) OR (rTSA) OR (reverse TSA) OR (reverse shoulder prosthesis) OR (reverse conversion) OR (reverse shoulder replacement) OR (reverse arthroplasty) AND (Glenoid Cavity[Mesh] OR glenoid types OR Walch glenoid OR glenoid bone loss OR glenoid bone defect OR posteriorly worn glenoid OR anteriorly worn glenoid OR superiorly worn glenoid OR bony defect OR posterior bone loss OR anterior bone loss OR superior bone loss OR glenoid deformity OR uncontained glenoid defect OR glenoid inclination OR glenoid retroversion OR Walsch type glenoid OR glenoid reconstruction OR glenoid deficiency OR glenoid bone deficiency OR glenoid erosion OR glenoid reconstruction OR glenoid dysplasia OR posterior glenoid deficiency OR glenoid morphology OR biconcave glenoid OR Favard Type glenoid) AND ("Bone Transplantation"[Mesh] OR "Transplantation, Homologous"[Mesh] OR "Transplantation, Autologous"[Mesh] OR autograft OR allograft OR structural allograft OR iliac crest autograft OR humeral head autograft OR structural glenoid grafting OR glenoid bone grafting OR primary glenoid bone grafting OR primary bone grafting OR femoral head allograft OR femoral allograft OR glenoid reconstruction OR structural bone graft OR glenoid augmentation OR bony increased offset reverse shoulder arthroplasty OR BIO RSA OR structural glenoid allograft OR glenoid reconstruction OR J-shaped bone graft OR bony glenoid augmentation)
Applied modifiers: last 10 years.

- Scopus

("reverse shoulder arthroplasty") AND ("glenoid bone defect") AND (allograft OR autograft)
Applied modifiers: last 10 years

- Epistemonikos

(title:((title:(reverse shoulder arthroplasty) OR abstract:(reverse shoulder arthroplasty)) OR (title:(reve* should* arthr*) OR abstract:(reve* should* arthr*))) OR abstract:((title:(reverse shoulder arthroplasty) OR abstract:(reverse shoulder arthroplasty)) OR (title:(reve* should* arthr*) OR abstract:(reve* should* arthr*))))

- Web of Science

#2 :ALL=((Arthroplasty, Replacement, Shoulder AND reverse) OR (Shoulder Prosthesis AND reverse) OR (reverse total shoulder arthroplasty) OR (RTSA) OR (reverse shoulder arthroplasty) OR (rTSA) OR (reverse TSA) OR (reverse shoulder prosthesis) OR (reverse conversion) OR (reverse shoulder replacement) OR (reverse arthroplasty))
#3: ALL=((Glenoid Cavity OR glenoid types OR Walch glenoid OR glenoid bone loss OR glenoid bone defect OR posteriorly worn glenoid OR superiorly worn glenoid OR bony defect OR posterior bone loss OR superior bone loss OR glenoid deformity OR uncontained glenoid defect OR Walsch type glenoid OR glenoid deficiency OR glenoid bone deficiency OR glenoid erosion OR glenoid dysplasia OR posterior glenoid deficiency OR Favard Type glenoid))
#4: ALL=((Bone Transplantation OR Transplantation, Homologous OR transplantation, Autologous OR autograft OR allograft OR structural allograft OR iliac crest autograft OR humeral head autograft OR structural glenoid grafting OR glenoid bone grafting OR femoral head allograft OR femoral allograft OR structural bone graft OR glenoid augmentation OR bony increased offset reverse shoulder arthroplasty OR BIO RSA OR structural glenoid allograft OR J-shaped bone graft OR bony glenoid augmentation))
#2 AND #3 AND #4
Applied modifiers: last 10 years

- Cochrane Database for Systematic Reviews
  -reverse total shoulder arthroplasty AND glenoid bone loss AND bone graft only provided 1 result. For this reason, we have decided to search the Cochrane Database using only the 1st concept (reverse total shoulder arthroplasty) in the hope that it will provide more results that we will screen for inclusion. The following search string provided the 4 mentioned results at the time the search was conducted (10th August 2022):

#1: Title Abstract Keyword = reverse total shoulder arthroplasty
#2: Title Abstract Keyword = RTSA
#3: Title Abstract Keyword = reverse shoulder arthroplasty
#4: Title Abstract Keyword = rTSA
#5: #1 OR #2 OR #3 OR #4
